# Acellular Mouse Kidney ECM can be Used as a Three-Dimensional Substrate to Test the Differentiation Potential of Embryonic Stem Cell Derived Renal Progenitors

**DOI:** 10.1007/s12015-016-9712-2

**Published:** 2017-02-27

**Authors:** Manpreet Sambi, Theresa Chow, Jennifer Whiteley, Mira Li, Shawn Chua, Vanessa Raileanu, Ian M. Rogers

**Affiliations:** 1 Women’s and Infant’s Health, Lunenfeld-Tanenbaum Research Institute, Mount Sinai Hospital and University of Toronto, 60 Murray St, Box 40, Toronto, ON M5T 3L9 Canada; 2 Department of Physiology, University of Toronto, Toronto, Canada; 3 Department of Obstetrics and Gynecology, Mount Sinai Hospital and the University of Toronto, Toronto, Canada

**Keywords:** Kidney, Extracellular matrix, Pluripotent cells, Stem cells, Organ culture, Decellularized kidney, Six2 +, Acellular

## Abstract

The development of strategies for tissue regeneration and bio-artificial organ development is based on our understanding of embryogenesis. Differentiation protocols attempt to recapitulate the signaling modalities of gastrulation and organogenesis, coupled with cell selection regimens to isolate the cells of choice. This strategy is impeded by the lack of optimal in vitro culture systems since traditional culture systems do not allow for the three-dimensional interaction between cells and the extracellular matrix. While artificial three-dimensional scaffolds are available, using the natural extracellular matrix scaffold is advantageous because it has a distinct architecture that is difficult to replicate. The adult extracellular matrix is predicted to mediate signaling related to tissue repair not embryogenesis but existing similarities between the two argues that the extracellular matrix will influence the differentiation of stem and progenitor cells. Previous studies using undifferentiated embryonic stem cells grown directly on acellular kidney ECM demonstrated that the acellular kidney supported cell growth but limited differentiation occurred. Using mouse kidney extracellular matrix and mouse embryonic stem cells we report that the extracellular matrix can support the development of kidney structures if the stem cells are first differentiated to kidney progenitor cells before being applied to the acellular organ.

**Electronic supplementary material:**

The online version of this article (doi:10.1007/s12015-016-9712-2) contains supplementary material, which is available to authorized users.

## Introduction

Ground breaking work demonstrated that rat neonatal kidney cells supported by the extracellular matrix (ECM) of an adult rat acellular kidney resulted in the restoration of kidney function [1]. This proof of principle study set the stage for using acellular kidney ECM as a substrate for three-dimensional cultures that are a better representation of the natural kidney environment compared to two-dimensional cultures. Acellular adult kidney ECM when combined with kidney cells can yield information on ECM-cell interactions representative of tissue repair since in many disease situations the ECM is exposed and is required to support new cells that migrate in from the periphery of the damaged area [2].

Importantly, determining the optimal decellularization protocol that maintains ECM integrity and determining the appropriate developmental stage of the therapeutic cells, whether it be a stem cell or a fully mature renal cell, are important for delineating the role of acellular ECM in regenerative medicine studies. Stem and progenitor cells have been tested as potential therapeutic cells for the treatment of kidney damage [3, 4]. Studies have also used pluripotent embryonic stem cells co-cultured with acellular kidneys and determined that the embryonic stem cells grew well but did not generate kidney progenitor or mature cells [5]. Another study using a kidney cell line demonstrated that cells adhere to the ECM thus proving that the ECM provided structural support for the cells. Whether the ECM directed differentiation of the kidney stem cell line towards mature kidney cell types was not shown as no kidney cell specific markers were used [6]. In our study we used acellular adult mouse kidney ECM combined with mouse stem or progenitor cells differentiated to three developmental stages; mesoderm, intermediate mesoderm and metanephric mesenchyme, to determine the mechanical and biological properties of the adult kidney ECM in regards to supporting cell differentiation and survival.

The most effective current therapeutic strategy for kidney failure is dialysis or transplantation. However, the average waiting time for a donor kidney is 3–5 years while the dialysis survival rate over 5 years is only 33% [7, 8]. In order to develop successful cell therapies we need a source of therapeutic cells for the treatment of kidney disease that are easily accessible, easy to grow, and efficiently differentiated. Developing therapeutic cells to treat kidney disease or the eventual production of whole kidneys for transplantation requires our ability to differentiate pluripotent cells into the different kidney cell types and induce three-dimensional organization. Decellularized kidneys can provide both structural and biological cues that promote progenitor cell differentiation and migration. Advantages to using natural ECM scaffold over artificial scaffolds include 1) the ECM scaffold has a distinct three-dimensional architecture that is difficult to replicate artificially, 2) the ECM scaffold houses location-specific proteins that guide adhesion, migration and differentiation, and 3) the vasculature ECM can be repopulated and utilized for the equal distribution of media and nutrients. Disadvantages include being able to procure suitable donor organs for decellularization. Organs removed during surgical procedures are biopsied for pathology rendering them inappropriate for whole organ decellularization. Organs donated for transplantation are in short supply and their availability is sporadic. We propose that porcine or bovine organs will become suitable alternative. Studies have determined that there is compatibility between cells and the ECM of different species [6], but more studies are required.

Adult ECM, that is supportive of mature cells and can influence tissue repair and homeostasis (reviewed in Theocharis. et al. [9],), is an ideal model to test the potential of pluripotent stem cells to respond to adult specific signals. Following the recent demonstration of the ability of the ECM from acellular lung to drive the differentiation of ES cell-derived endoderm to mature lung cells led us to investigate if it is possible to decellularize kidneys while maintaining the same level of mechanical and biological support we observed with the lung model [10]. Building on previous published studies we tested different methods of decellularization including soak decellularization of thick tissue sections, as well as perfusion decellularization through either the vasculature or the ureter of mouse kidneys. We also tested different detergents and different treatment times. We are able to demonstrate that the adult mouse kidney acellular ECM can support the growth and maturation of mouse embryonic stem cell derived metanephric mesenchyme progenitor cells.

## Methods

### Ethics and Approvals

All mouse work was approved by the Animal Care Committee at the Toronto Centre for Phenogenomics at Mount Sinai Hospital, Toronto, Canada.

### Kidney Decellularization

Multiple methods were used to decellularized mouse kidneys. Either whole mouse kidneys or thick transverse kidney sections- approximately1000μm- cut using a Leica Vibratome or razor blade, were decellularized under constant perfusion of the decellularization solution. For whole kidneys, **t**he renal artery was cannulated using a blunt ended 30-gauge needle and held in place with 6–0 sutures. The kidney-needle complex was then attached to surgical tubing with an internal diameter of 3/32″ using a luer lock. The ureter was cannulated using surgical tubing and held in place with sutures. The cannulas were maintained and used for recellularization. A peristaltic pump was used to achieve a controlled continuous flow of liquids through the kidney. 0.1% SDS at flow rates of 0.2 and 0.4 ml/min for 12, 24, 48 or 72 h, followed by +/− 1 h wash with 0.1% Triton X-100 then a 24 h wash with 0.1% PenStrep/PBS. The whole acellular kidney could be sectioned at this point for cell co-culture studies.

Alternatively, kidney sections could be cut first then decellularized. Thick sections were cut and treated with 0.1% SDS using peristaltic pump (0.4 ml/min) to provide for a constant flow of the SDS solution over the sections for 24 h, followed by wash in PBS (O/N) and PBS/PenStrep for 1 h. The constant flow resulted in the removal of cellular debris.

Two alternative detergents were tested: 0.1% Triton X100 for 24–72 h or 0.4% Sodium Deoxycholine for 24–72 h +/− 90 U/ml benzonase for 2 h, were used to decellularize the kidney at the same flow rate and times as for the SDS protocol. If required, decellularized kidneys were stored at 4 °C for <5 days prior to recellularization.

### Kidney Recellularization

A 30-gauge needle was used to overlay and inject cells into acellular kidney sections. The repopulated kidney sections were cultured submerged in 10%FBS/DMEM/Antibiotic. For whole kidney recellularization the cells were introduced through the ureter while the kidney was under negative pressure. The negative pressure was applied using a vacuum hooked up to a chamber made from either a 50 ml plastic tube or a glass bottle. The vacuum was maintained just for cell loading. Whole kidneys were fed by perfusing 3–5 ml of medium through the vasculature daily or continuous perfusion with medium. Sections were maintained with 50% medium changes daily. All recellularized kidneys were incubated at 37 °C, 5% CO_2_.

### Tissue Processing and Immunochemistry, Hematoxylin and Eosin (H&E) Staining

All tissues were fixed in 10% neutral buffered formalin (NBF) and processed as described [11].

Primary antibodies: All used at 1:100: β-Catenin (Santa Cruz sc7199), Brachyury (BRY) N-19 (Santa Cruz sc-17,743), HNF3β respectively (Millipore 07–633 and Santa Cruz SC9187), OCT-3/4 (Santa Cruz sc-8628), PAX-2 (Covance PRB-276P), SIX-2 (ProteinTech, 11,562-AP), SOX-17 (R&D Systems AF1924), WT-1 (Abcam ab89901). Keratin-16 (KSP) (Novus Biologics NB159248), Laminin (Abcam ab11575), Fibronectin (Abcam ab23750), Collagen IV (Abcam ab6586), HSPG (Abcam ab2501), Vimentin (Santa Cruz sc7558), Podocin (Sigma P0372), panCytokeratin (Sigma p2871).

Cells were stained with DAPI to detect nuclei and the signal was preserved using DABCO. Images were analyzed on the Leica DM IL microscope and images were taken with a Hamamatsu ORCA-03G camera.

#### Quantification of Cells

Three fields of view were randomly chosen/well. All DAPI stained cells and desired antibody positive stained cells were counted per field.

### Isolation of Day 12.5 Renal Progenitor Cells

Day 12.5 embryos were retrieved from pregnant dams and all extraembryonic tissues were removed. The head was removed and the liver, lungs and digestive system were gently removed revealing the developing kidneys. Kidneys were collected and washed in cold PBS, followed by gentle trituration to obtain a single cell population.

### ES Cell Culture

B6 e-GFP and BRY-GFP mouse ES cell lines [12] were grown on mitomycin C mitotically inactive mouse embryonic fibroblast (MEF) feeder layers. Cells were passaged every other day using 0.25% trypsin (Life Technologies) and split at a ratio between 1:10 and 1:20. Cells were incubated at 37 °C, 5% CO2. Mouse ES cells were cultured in standard mouse ES medium supplemented with LIF (1000 U/mL, Life Technologies). Mouse ES medium was made with 500 mL DMEM (GIBCO), 20% FBS (GIBCO), 0.5× PenStrep (GIBCO), 100 μM β-mercaptoethanol (Sigma), 1 mM Na pyruvate (Sigma), 10 μM non-essential amino acids (GIBCO), 2 mM GlutaMAX™ (Invitrogen).

#### Differentiation Protocol

Mouse ES cells from a 90% confluent plate were used for differentiation. Cells were washed with PBS and trypsiznized with 0.25% trypsin and incubated at 37 °C for 5 min to allow cells to detach from feeders and plate. Cells were centrifuged at 1200 rpm for 4 min at 10 °C and supernatant was removed and cells were resuspended in mesoderm differentiation medium: Variations of the medium used are described in detail in the results section:

##### Final Protocol

- Stage 1: Mesoderm: 200,000 cells/cm^2^ on gelatin plates two days with Serum Free Medium (SFM): DMEM/F12 + 0.5% serum replacement, Activin A 30 ng/mL to induce mesoderm.
- Stage 2: Intermediate mesoderm: Two days in serum containing medium (SCM): DMEM/high glucose, 4% FBS, 10 μM Y-27632 and 30 ng/ml Activin A, 100 nm RA to induce PAX2+ intermediate mesoderm.
- Stage 3: Mesenephric mesenchyme: Followed by 3.5 days in Modified SCM (4% serum in DMEM) supplemented with FGF2 at 0.1 ng/ml to induce Six2+ cells. Change medium at day 1.5. Wash 1× PBS between media changes.

### Components

**Table:** 

Name of product	Company	Catalogue number
2-Mercaptoethanol	Sigma-Aldrich	M7522
Accutase™	Innovative Cell Technologies	AT-104
Activin A	R&D Systems	338-AC
Agarose	Bioshop	AGA001
Albumin from Bovine Serum	Sigma-Aldrich	A-2153
BMP-4	Invitrogen Life Technologies	PHC9534
BMP-7	Invitrogen Life Technologies	PHC9544
DABCO	Sigma-Aldrich	D27802
DAPI	Sigma-Aldrich	D9542-IMG
DMEM	GIBCO	11960–044
DMEM/F12	Invitrogen	11039–021
DMEM/High Glucose	In house	NA
Fetal Bovine Serum	In house	
FGF2	Invitrogen	13256–029
GDNF	R&D Systems	212-GD-010
GlutaMAX™	Invitrogen Life Technologies	35050–061
KO/DMEM	GIBCO	10829–018
LIF	In house	
LY294002	Promega	V1201
Na pyruvate	Invitrogen	11360–070
NEAA	Invitrogen	11140–050
PenStrep	GIBCO	15140–122
RA	Calbiochem	554720
Serum replacement	Invitrogen	10828–028
Stauprimide	Tocris Biosciences (R&D Systems)	154589–96-5
TrypLE™	Invitrogen Life Technologies	12605010
Y27632 (ROCK inhibitor)	Cederalane	688000

### RT-PCR

RNA was isolated using Qiagen RNA Isolation Kit and cDNA was made. Primers sequences were as follows:

- *Pax-2* (5’-AGGGCATCTGCGATAATGAC-3’ and 5’- CTCGCGTTTCCTCTTCTCAC-3’)
- *Wt-1* (5’-ACCCAGGCTGCAATAAGAGA-3’ and 5’- GCTGAAGGGCTTTTCACTTG-3’)

PCR was performed with a denaturation at 94 °C for 2.5 min and 30 cycles for 94 °C for 30 s, annealing at 58 °C for 1 min, 72 °C for 1 min and 72 °C for 10 min for final extension.

- *Emx2* (5’-CCGAGAGTTTCCTTTTGCA-3’ and 5’-GCCTGCTTGGTAGCAATTC-3’)
- *Eomes* (5’-GGCAAAGCGGACAATAACAT-3’ and 5’-AGCCTCGGTTGGTATTTGTG-3’)
- *CD56* (5’-GATCAGGGGCATCAAGAAAA-3’ and 5’-CTATGGGTTCCCCATCCTTT-3’)
- *Hoxd11* (5’-ACTCCAGGCAAACGAGAGAA-3’ and 5’-GCTGGTTGGAACAAGCAAAT-3’)
- *Sall1* (5′-CCCCATCCCTATTAGCCATT-3’ and 5’-AGAGTACTGTTGCCCGCTGT-3’).

Annealing at 56 °C for 1 min

- *Mesp1* (5’-CCTTCGGAGGGAGTAGATCC-3’ and 5’-AAAGCTTGTGCCTGCTTCAT-3’).

Annealing at 54 °C for 1 min

- *ß-Actin*(5’-CATCCGTAAAGACCTCTATGC-3’and5’-AGAAAGGGTGTAAAACGCAGC-3’)
- *Gsc* (5’-AACGCCGAGAAGTGGAACAA-3’ and 5’-AGGCAGGGTGTGTGCAAGT-3’)
- *Cited1* (5’-ATGCCAACCAGGAGATGAAC-3’ and 5’-AAGCTCATTGGCTCGGTCTA-3’)

Annealing at 60 °C for 1 min.

#### Cytokine Array

Mouse proteome array kit from R and D systems (ARY015) was used to detect cytokines on decellularized extracellular matrices. User manual was followed to conduct protocol. Arrays were exposed to X-ray film for 1, 2, 3, 5, 10 and 15 min. Pixel density was measured with the ImageQuant program and charts were made using Microsoft Excel. Protein networks were determined using STRING v.10 (Search Tool for the Retrieval of Interacting Genes/Proteins). Three independent experiments were done and each one was done in triplicate to obtain the mean pixel density.

#### Scanning Electron Microscopy

For SEM samples were fixed in 2.5% glutaraldehyde in 0.1 M phosphate buffer pH 7.4 and post-fixed in osmium tetroxide, dehydrated in an ascending series of ethanols and critical point dried. All samples were then mounted on aluminum stubs using double-sided carbon tape and rendered conductive with a thin coat of gold palladium using a sputter coater and examined and photographed in a field emission scanning EM using a Phillips FEI XL30 (30 kV Scanning Electron Microscope).

## Results

### Preparation and Characterization of biologically Active Extracellular Matrix

To maximize the biological activity of the ECM, it was critical to determine the minimal decellularization treatment that is required to remove cells but retain the ECM architecture and embedded growth factors that would support cell survival, adhesion and differentiation [16, 17]. Whole mouse kidneys or transverse kidney sections were decellularized in a solution of 0.1% SDS or 1% Triton X-100 or 0.4% Sodium deoxycholate by continuous perfusion. Intact mouse kidneys were perfused using whole organ retrograde perfusion through cannulation of the renal artery. We also attempted perfusion decellularization through the ureter. The artery cannulation allowed for the continuous flow of decellularization solution through the kidney without causing a build up of pressure when flow rates of 0.2–0.4 ml min^−1^ were used. Triton X-100, a non-ionic detergent had been demonstrated to maintain ECM proteins better than SDS, but it was not an effective decellularization agent for dense organs such as cardiac tissue and kidney [13]. In our hands Triton X-100 did not result in decellularization. Even after 48 h of continuous perfusion there was no indication that any decellularization was occurring. Sodium deoxycholate was able to lyse cells but much of the cellular debris remained in the kidney even with constant perfusion. Treatment with benzonase did not improve the clearance of the cell debris and DNA (Supplementary Fig. 1). Thus, SDS was the only treatment that resulted in removal of cellular proteins and DNA. We also attempted to deceullarize using a cannulated ureter instead of the vasculature. Perfusion through the ureter using 0.1% SDS at 0.4 ml min^−1^ did not result in any noticeable decellularization even after 48 h of continuous perfusion, indicating that the blind ends of the bowman’s capsule results in back pressure that prevented proper decellularization.

**Fig. 1:**
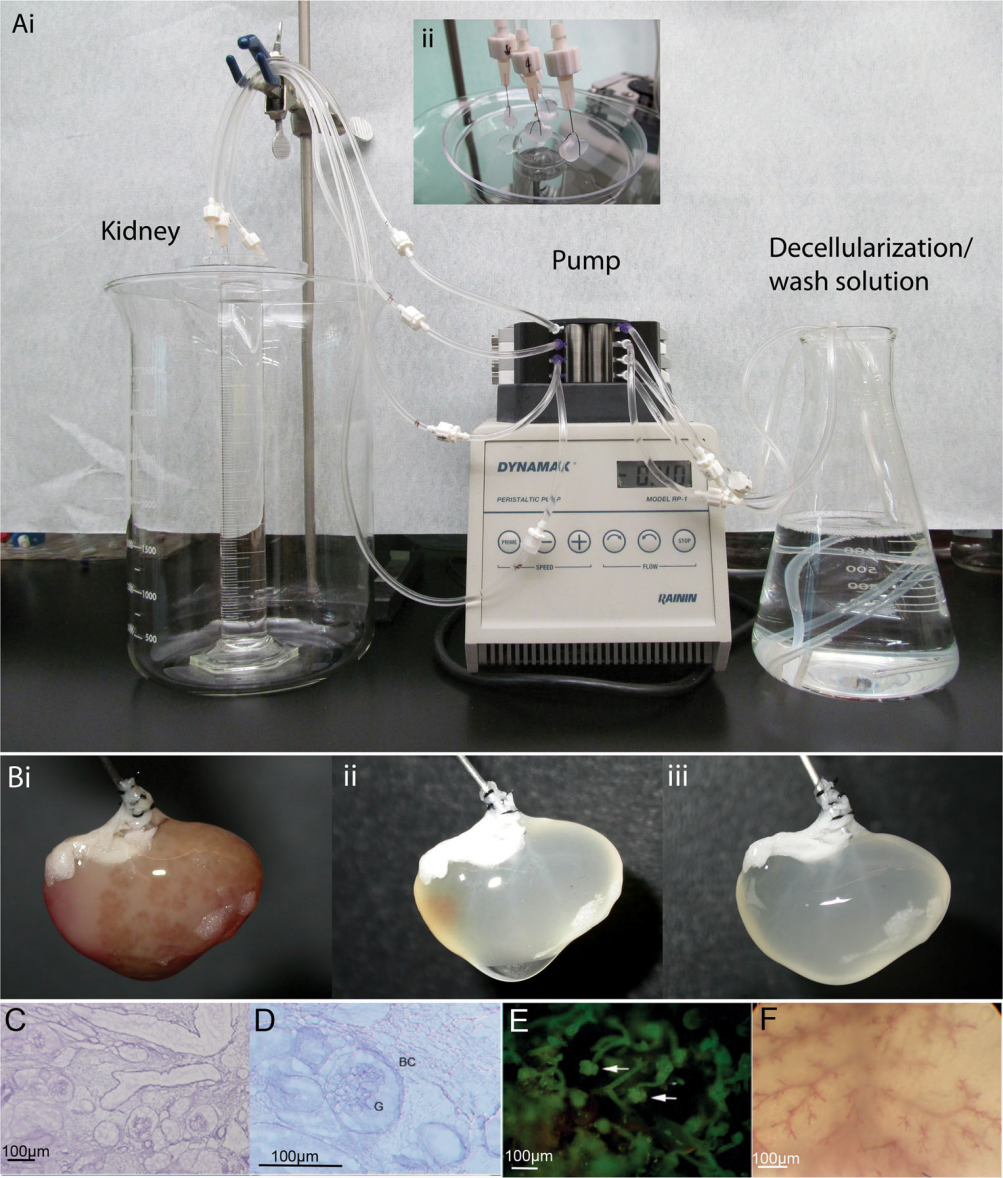
Adult mouse kidneys were decellularized with 0.1% SDS for 72 h at a flow rate of 0.4 ml/min. The kidney capsule interferes with soaking methods so a continuous flow system was used for whole organ decellularization. **Ai**) Solutions are pumped through the vasculature of the cannulated kidney using a peristaltic pump. Aii) The kidney is kept at the air-liquid interface during de-cellularization. This helps to move cellular debris away from the kidney. **B**) De-cellularization progression of an adult mouse kidney. i) time = 0 h, ii) time = 24 h and iii) time = 72 h. (**C**) H&E staining showing intact glomeruli (G) and Bowman’s capsules (BC) at 20× and (**D**) 40×. (**E**) Progressive vascular corrosion casting of a mouse kidney show multiple intact glomeruli (*arrow*) and (**F**) intact vasculature. Magnification bar = 100 μm

Using the whole organ retrograde perfusion with 0.1% SDS, most whole kidneys were fully decellularized at 24–48 h but in some cases residual cellular debris including DNA remained. Decellularization for 72 h followed by 24 h of PBS perfusion was optimal for achieving fully decellularized kidney (Fig. 1 a, b). H&E staining post decellularization revealed that kidney microstructures maintained intact (Fig. 1c, d) and corrosion casting and dye infusion revealed that the vasculature, including capillaries, was not damaged (Fig. 1e, f). Interestingly, the liquids perfused through the acellular vasculature did not leak through the ECM as expected. This has also been observed by others and is likely due to the release of tension on the ECM structure due to the loss of cells, causing the ECM protein strands to contract thus forming a barrier [14].

**Fig. 2:**
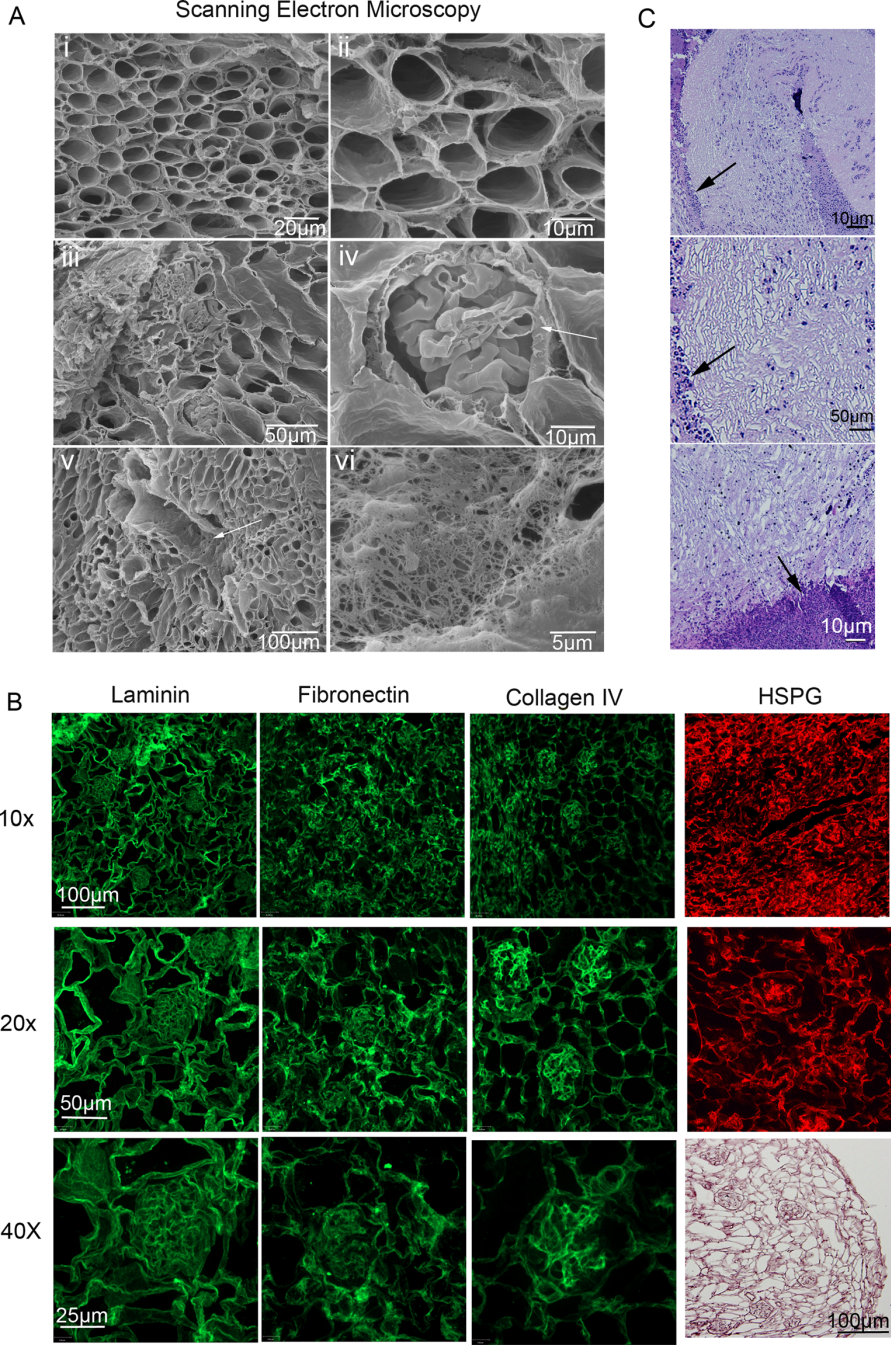
Characterization of decellularized adult kidney matrices. Scanning electron microscopy of a decellularized kidney. (**Ai,ii**) Low and high magnification of the kidney cortex depicts the large array of nephron tubules. High magnification of intact decellularized tubules demonstrated that all cells and cellular debris were removed while keeping the ECM intact. (Aiii, iv) Cross sections of glomeruli and Bowman’s capsules can be observed. At the plane of the tissue cross section the glomerular basement membrane can be seen (*arrow*). Despite the thinness, the glomerular basement membrane remained intact. (Av, vi) A cross section of a renal artery (arrow) and high magnification of the internal structure of the same renal artery showing the elastin fiber structure remained intact. (**B**) H&E staining shows no cells remain on the ECM, and the architecture of the kidney matrix remains intact (lower right). Laminin and Fibronectin were expressed ubiquitously in the decellularized kidney matrix indicating that the structural and biological integrity of a decellularized matrix is intact. Collagen IV and HSPG demonstrate regions of high and low protein expression with the strongest expression in the glomerular ECM. This is best illustrated at the higher magnification. (**C**) To test antigenic potential of the ECM an adult C57Bl/6 mouse kidney was decellularized for 24 h and transplanted into a CD1 mouse by folding the abdominal fat around the kidney and autopsied after 14 days. Note the minimal cell infiltration and the decellularized kidney morphology remained unchanged. The border between the acellular kidney and the fat pad is denoted by arrows

Scanning electron microscopy (SEM) and immunofluorescence (IF) were utilized to observe the details of the acellular kidney ultrastructure (Fig. 2a,b). Intact tubules, blood vessels and glomeruli could all be observed. Breaks in the capillary tufts were at the plane of sectioning and the extremely thin capillaries were not damaged by the flow of decellularized solutions (Fig. 2Aiv-arrow). A large blood vessel still contained fine elastin fibers after decellularization further attesting to the retention of fine ECM structures after decellularization (Fig. 2Av-arrow,vi).

Sections of decellularized kidney were stained with H&E to confirm complete decellularization. Then adjacent sections were analyzed by IF and are displayed at three different magnifications to show the broad staining pattern and the region specificity (Fig. 2b). Laminin and Fibronectin demonstrated ubiquitous staining, while Collagen IV and Heparan Sulfate Proteoglycan (HSPG) were uneven with more protein observed in the glomeruli. HSPG is a basement membrane protein that sequesters and regulates FGF, BMP and WNT, and promotes interactions between cells and the ECM. Fibronectin, Laminin and Collagen IV are ECM proteins involved in cell adhesion and cell migration [15, 16]. The non-uniformity of the HSPG and Collagen IV expression could be due to the action of the SDS decellularization reagent or kidney specific localization. IHC with the fluorescent secondary antibody only, along side the primary antibody plus secondary antibody staining, bright-field pictures and DAPI of normal mouse kidneys and decellularized mouse kidneys are in Supplementary Fig. 2. Complete decellularization is demonstrated by the complete absence of DNA (DAPI stain negative). ECM proteins, Laminin, Fibronectin and Collagen IV are present in both untreated and decellularized mouse kidneys. Secondary antibody only staining demonstrates the results is due to specific staining of the primary antibody.

### Decellularized Kidneys Are Not Immunogenic

Although the acellular kidney can be used as a 3D substrate for tissue culture to test properties of individual kidney cells, ultimately the goal is to use acellular organs and stem cells to produce a whole functional organ for transplantation. Potential sources for acellular kidney substrates include human donor kidneys that are not suitable for transplantation due to HLA mismatching, damage or disease. An alternative source is bovine or porcine kidneys. For any source to be practical for use in transplantation, the acellular kidney must not be immunogenic. We tested this by decellularizing a C57Bl/6 mouse kidney (H2K^b^) using our SDS perfusion system and then transplanted a whole acellular kidney into the abdomen of CD1 (H2K^d^) mice by attaching the kidney to the fat pad. After two weeks the fat pad and kidney were excised. No morphological damage was observed indicating that mouse ECM proteins are not antigenic and acellular organ-stem cell constructs can be used to produce organs for transplantation (Fig. 2c arrows indicate kidney-fat pad border).

### Protein Array Analysis of the Acellular Kidney

The goal of decellularization was to retain ECM proteins post decellularization including focal adhesions containing cytokeratins and integrins. We hypothesized that the retention of bioactive proteins, such as HSPG, would aid in the repopulation and differentiation of stem and progenitor cells as observed in studies of acellular lungs repopulated with ES cell-derived endoderm [10]. To further assess the proteins that were present in the decellularized ECM, a protein array of 53 proteins was employed. It was determined that the ECM possessed 22 of the 53 proteins tested. The role of the 22 proteins is summarized in Table 1.

**Table 1:**
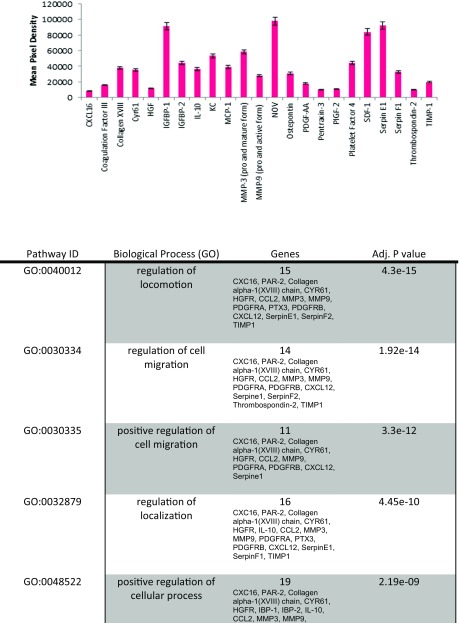

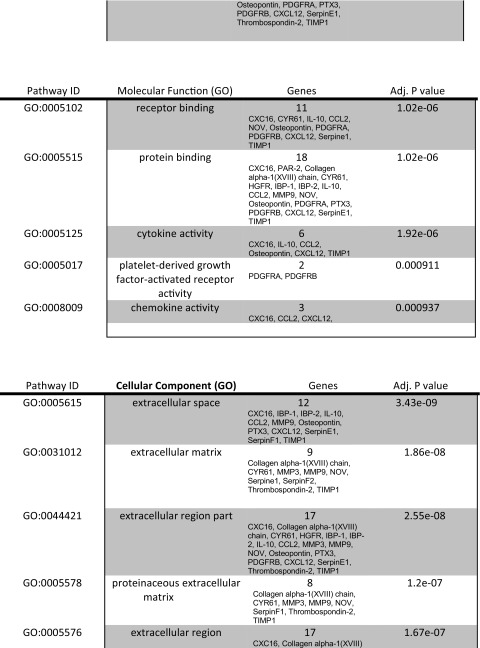

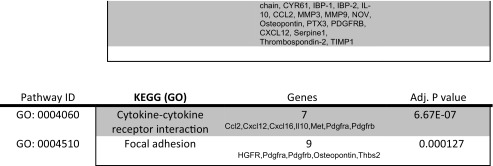
Analysis of ECM proteins by protein array. The decellularized adult mouse kidney ECM contains proteins required for cell differentiation, survival and ECM remodeling. Protein array of acellular adult kidneys demonstrated the expression of 22 proteins. Their roles and interactions are listed

**Fig. 3:**
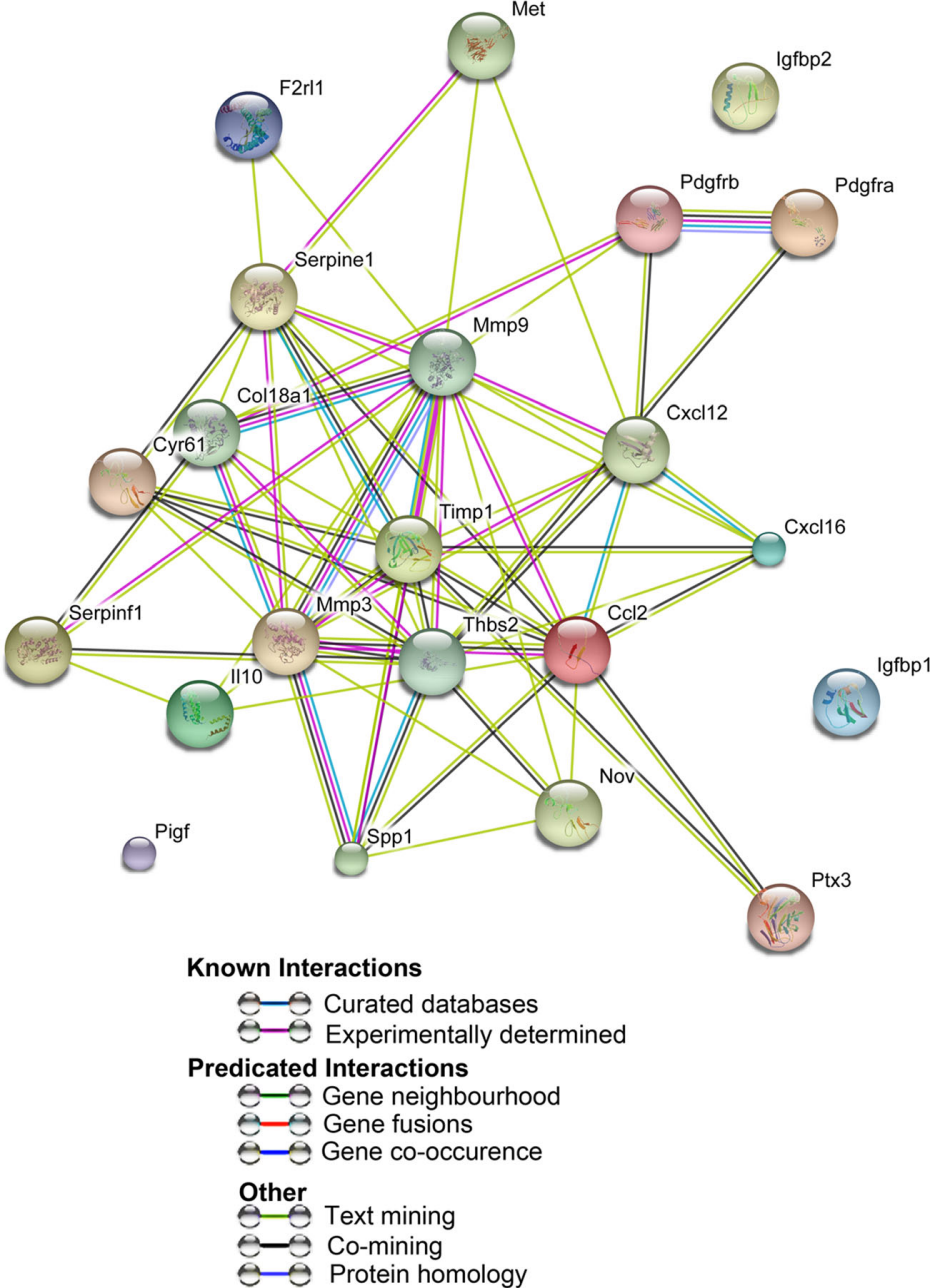
Proteins expressed in acellular kidney ECM are enriched for tissue repair and regeneration. Network visualization of GO terms enriched in the set of proteins expressed in acellular kidney ECM. Breakdown of the different pathways are listed in Table 1. Pathways that reached statistical significance are focused on cell migration, proliferation and ECM interactions that are involved in cell and tissue repair. Interactions were derived from various databases listed in the index

In order to assess possible interactions between the 22 highly expressed proteins we used STRING (Search Tool for the Retrieval of Interacting Genes/Proteins) [17] (Fig. 3). STRING identified a number of functional enrichments. Many of the biological process identified were involved in cell migration including cell locomotion and localization. Molecular functions that were identified were related to biological processes and included receptor binding, cytokine and chemokine activities. Cellular component pathways identified, not surprisingly, were grouped together under extracellular matrix components. KEGG (Kyoto Encyclopedia of Genes and Genomes) pathways identified both focal adhesion and cytokine-cytokine receptor signaling pathways. Taken together, the different processes identified were related to tissue regeneration and repair. Induction of embryo development processes did not reach significance suggesting the decellularized kidney ECM has a stronger role in tissue regeneration than organogenesis. This may also be a function of GO term definitions as ‘embryo developmental’ processes include the whole embryo and not just kidney organogenesis.

### Repopulating Acellular Kidneys

Acellular kidney sections or whole kidneys were combined with cells to determine cell distribution and survival. Thick sections of acellular kidney were either injected with cells or overlaid with cells. Both protocols resulted in large areas of the ECM being repopulated. The route for repopulating the whole decellularized kidney required some consideration. Although cells were capable of moving through the ECM under normal organ activity [18], there was little evidence that this occurred with high efficiency with decellularized organs. Therefore, introducing nephron-specific cells through the ureter was required, instead of using the vasculature and expecting the cells to migrate across the ECM. The renal artery was cannulated with a needle while the ureter was cannulated with a flexible tube. Repopulation of the whole kidney through the ureter was done by placing the kidney in a vacuum chamber that set up a negative pressure gradient in the kidney resulting in the even distribution of cells into the cortical region. Renal epithelial cells or stromal cells were used to test repopulation of a whole acellular mouse kidney (Supplementary Fig. 3). Although cell repopulation of whole acellular kidneys worked well, acellular kidney sections were easier to use and allowed for the analysis of multiple parameters using less kidney tissue and fewer animals. Sections were used throughout this study.

### Adult Acellular Kidney ECM Has a Limited Impact on the Differentiation of ES Cells and Intermediate Mesoderm

The kidney is composed of over thirty different cell types. Attempting to generate each of the required mature cells in vitro, followed by placing them at the correct coordinates within the acellular kidney would be impossible. We hypothesized that the ECM of the acellular kidney maintains localized bioactive proteins that may guide the differentiation of stem or progenitor cells, cell migration, homing and cell adherence. In order to test the differentiation induction capacity of the ECM, we generated and used undifferentiated mouse ES cells, ES cell-derived BRY+ mesoderm, PAX2+ intermediate mesoderm (IM) and SIX2+ metanephric mesenchyme (MM) stage cells that were co-cultured with decellularized kidney ECM or on gelatin coated plates as negative controls. Monolayers of ES cells grown without LIF for four days on gelatin plates acted as controls for spontaneous differentiation.

**Fig. 4:**
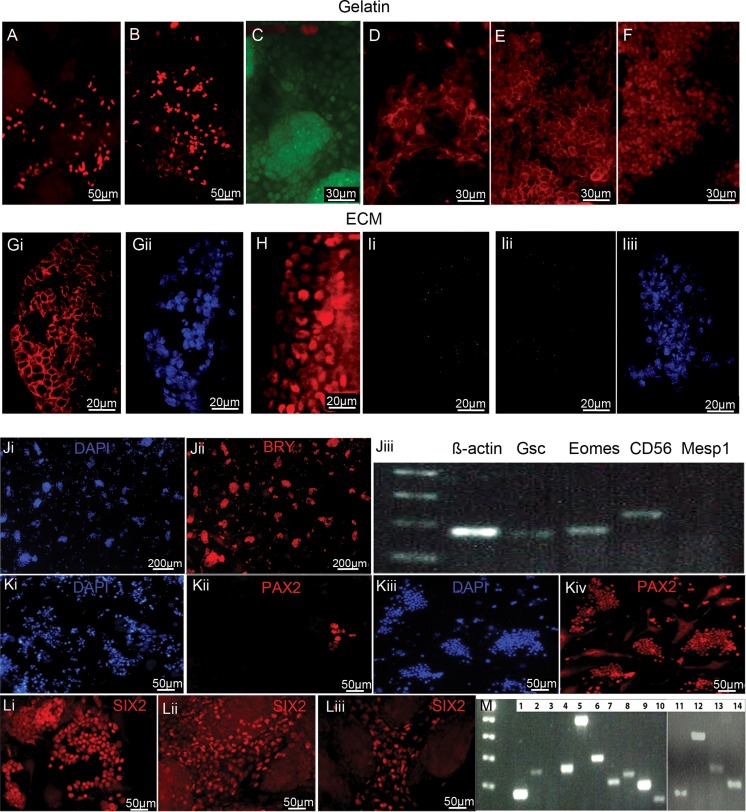
The acellular kidney matrix can influence the differentiation of ES cells to mesoderm only, but mature kidney cells can be obtained when metanephric mesenchyme are used to repopulate the acellular matrix. When cells were grown for 4–10 days on gelatin coated culture plates in the absence of the ECM, spontaneous differentiation occurred resulting in endoderm cells (**A**) SOX17+ and (**B**) HNF3β+, and mesoderm (**C**) BRY+ cells. Kideny specific proteins (**D**) Cytokeratin (**E**) ß-Catenin were present but the kidney progenitor marker PAX2 and the mature kidney marker KSP were negative on the gelatin grown cells. (**F**) OCT4+ cells indicate many of the cells did not undergo differentiation. In contrast, ES cells grown on adult kidney ECM showed evidence of directed differentiation of the ES cells as evidenced by only mesoderm derivatives being present, specifically kidney precursor markers (**Gi**) ß-Catenin (Gii: DAPI) and (**H**) PAX2. (Ii) Endoderm specific protein SOX17 was negative. (**Iii**) KSP was negative (Iiii: DAPI). ES cells were differentiated in specialized medium to obtain BRY+ mesoderm. BRY expression in media conditions supplemented with Activin A, BMP-4 and FGF2 in varying concentrations and combinations were determined by IF. The total number of cells and the number of BRY positive cells were scored by counting three random fields per well (*n* = 4 wells). (**Ji, ii**) the relative efficiency of mesoderm production was 80% + using our optimal protocol: Serum Free Medium (SFM): DMEM/F12 + 0.5% serum replacement containing Activin A 30 ng/ml for 2 days resulted in 80% BRY+ cells. (Jiii) RT-PCR confirmed commitment to mesoderm cell lineage and its derivatives as shown by expression of *Gsc*, *Eomes* and *CD56*. *Mesp1*, a marker of mesoderm regionalization was negative indicating the cells are at an early mesoderm stage. *ß-actin* (load control). (**K**) ES cells were first differentiated to BRY+ mesoderm then differentiated to intermediate mesoderm (PAX2+). Different conditions were tested to optimize cell survival and differentiation. (Ki,ii) Activin A 30 ng/mL + RA 100 nM in minimal medium (RPMI/0.5% SR) which minimizes exposure of the cells to unknown proteins, resulted in some PAX2+ cells but high cell death. (Kiii,iv) Changes in serum and growth factor combinations lead to a high frequency of PAX2+ cells and high cell survival using DMEM/high glucose, 4% FBS, 10 μM Y-27632 and 30 ng/ml Activin A, 100 nm RA. In order to obtain MM SIX2+ cells (**L**) PAX2+ cells were grown in varying combinations and concentrations of FGF2 with or without BMP-7. All conditions showed SIX2 expression, however, none yielded significantly more SIX2 than (Li) FGF2 at 0.1 ng/ml or (Lii) FGF2 at 0.1 ng/ml + BMP-7 at 5 ng/ml. As a comparision (Liii) increasing BMP7 to 25 ng/ml resulted in less SIX2+ cells. (**M**) PCR confirmed ES cell derived SIX2+ MM cells expressed other metanephric mesenchyme specific genes: 1) *ß-actin* (load control) 2) *Pax2* 3) blank well 4) *Wt1* 5) *Cited1* 6) *Emx2* 7) *Hoxd11* 8) *Hoxb7* 9) *Sall1* 10) *Sall4* 11) *ß-actin* (load control) 12) *Wt1* 13) *Cited1* 14) *Eya1*

Undifferentiated mouse ES cells survived and proliferated on both gelatin plates (control) and the acellular kidney. ES cell grown on gelatin-coated plates (minus LIF) underwent spontaneous differentiation as expected, producing SOX17+ and HNF3ß + endoderm and BRY+ mesoderm (Fig. 4a-c). Two kidney proteins, Cytokeratin and ß-Catenin, were also produced from the spontaneously differentiating cells (Fig. 4d, e). Oct4 pluripotent cells were still present mainly in areas of cell colonies (Fig. 4f). In contrast, ES cells grown on the decellularized adult kidney sections (minus LIF) were positive for only mesoderm as they expressed ß-Catenin and PAX2 (Fig. 4g,h). The cells did not differentiate into endoderm as the cells were negative for SOX 17 (Figure Ii). Despite the ability to make mesoderm, more mature kidney cells did not form nor did mature structures such as tubules since the cells were negative for KSP (Figure 4Iii-iii). This observation suggested that the kidney ECM may retain bioactive proteins that preferentially supported the differentiation of ES cells to mesoderm over endoderm but not to mature kidney cells. Lastly, we heat-treated the ECM at 55 °C for 10 min and applied ES cells. There were few cells observed and all were negative for mesoderm, IM and kidney proteins indicating the ECM provides some bioactive support for differentiation to mesoderm that is heat sensitive (data not shown).

The ability of the ECM to support ES cell differentiation to mesoderm but not endoderm or mature kidney cells prompted us to investigate what would occur if mesoderm, PAX2+ IM or SIX2+ MM cells were co-cultured with the ECM instead of pluripotent ES cells. Despite being able to generate mesoderm the kidney ECM could not support further differentiation but it was possible that the adult ECM was more supportive of later stage differentiated cells, as dedifferentiation and re-differentiation is a hallmark of kidney repair [19]. In order to determine the extent that the kidney ECM can support further kidney differentiation or cell proliferation and growth of different stages of kidney development we applied mesoderm, PAX2+ IM or SIX2+ MM cells to acellular kidney sections. A protocol to generate mouse ES cell-derived mesoderm, IM and MM cells using cell monolayers in defined differentiation media was developed. Since differentiation efficiency varies with different ES cell lines and protocols, the optimal concentrations of growth factors, serum and inhibitors for mouse B6 eGFP and BRY-GFP ES cell differentiation was determined empirically.

FGF2, Activin A and BMP4 have critical roles in mesoderm induction during gastrulation. Hence, different combinations and concentrations of these three factors were used on monolayers of ES cells in Serum Free Medium (SFM). The following conditions were tested: DMEM/F12 + 0.5% serum replacement media containing Activin A (5, 30 or 50 ng/ml) +/− FGF2 (0.01–50 ng/ml) and +/− BMP4 (5–50 ng/ml). Activin A alone at all three concentrations resulted in BRY+ cells. The addition of high concentrations of FGF2 resulted in high levels of cell proliferation, the retention of ES-like colonies and low BRY expression (<1%). FGF2 at 0.01 and 0.1 ng/ml with all concentrations of Activin A showed no improvement over Activin A alone. The addition of BMP4 to all of the growth factor combinations had a mild antagonistic role in our system consistent with studies indicating a central role of SMADs in balancing BMP4 and Activin A signaling during mesoderm patterning [20]. Activin A alone at 30 ng/ml was sufficient to differentiate >80% of the ES cells to Bry + cells (Fig. 4Ji-ii). We hypothesize that in monolayer cultures the normal cell-cell interactions that occur during embryo development are absent and this accounted for the reduced role for FGF2 and BMP4 in monolayer differentiation cultures. The Bry + cells also expressed markers of mesendoderm and mesoderm, namely *Gsc*, *Eomes* and *CD56*. *Mesp1* was negative indicating that the mesoderm was not yet specified into lateral, intermediate or paraxial mesoderm (Fig. 4Jiii). In order to test the potential of the kidney ECM to differentiate BRY+ cells to mature kidney cells, the BRY+ cells were harvested from the plates and layered onto acellular kidney ECM as described for ES cells. The majority of cells did not survive disassociation and replating despite the use of various disassociation agents and ROCK inhibitor and the few surviving cells did not attach to the ECM.

We next determined if PAX2+ IM would survive the dissociation and repopulating process better than early stage mesoderm. Retinoic acid (RA) has an important role in patterning the developing embryonic tissue into lateral, intermediate and paraxial mesoderm [21–23]. Activin A also continues to be important for intermediate mesoderm patterning during embryogenesis [24] and BMPs establish a concentration gradient that patterns the dorso-ventral axis. We tested different concentrations of these growth factors in order to induce PAX2. Concentrations of Activin A (5 ng/ml to 50 ng/m) with or without BMP4 (50 ng/ml), BMP7 (50 ng/ml) and RA (100 nM) in SCM were tested. Initial plating confluency and plate coating were also taken into consideration.

Serum or serum replacement was tested at concentrations ranging from 0.5% to 10% with or without ROCK inhibitor, a potent inhibitor of apoptosis, in order to optimize cell survival and differentiation rates [25]. Cell loss during differentiation meant maximizing the initial cell density but not at the risk of disrupting the cell monolayer, which was vital to uniformly differentiated cells. Protocols using embryoid bodies rely on cell-cell signaling to induce and maintain differentiation while using monolayers of cells as done here, requires the determination of the appropriate cell density that induces differentiation but does not overcrowd the cells as they keep proliferating. Cells were plated at a density of 50 K, 10 K or 2.5 K per cm^2^ on gelatin plates at the start of the differentiation protocol. Low cell density resulted in few PAX2+ cells (<5%) (Fig. 4Ki-ii). Although the majority of cells survived they did not differentiate indicating that cell-cell signaling is important for the differentiation process and thus higher cell densities are required. Starting with 50 K cells/cm^2^ for stage 1 (mesoderm) resulted in a dense monolayer of BRY+ cells that yielded PAX2+ cells at subsequent differentiation stages. The final conditions that yielded the highest frequency of PAX2+ cells (80%+) was 50 K cells/cm^2^ on gelatin plates for 2 days with Activin A 30 ng/mL to induce mesoderm followed by 2 days in PAX2 induction conditions comprised of DMEM/high glucose, 4% FBS, 10 μM Y-27632 and 30 ng/ml Activin A, 100 nm RA (Fig. 4Kiii-iv).

The transfer of PAX2+ cells to the acellular kidney gave the same results as the BRY+ cells. The PAX2+ IM cell survival was low after disassociation even when ROCK inhibitor was added suggesting that at the IM stage cell-cell contact is important for cell survival. As an alternative strategy, ES cells were directly plated on acellular kidney sections and grown in conditions described above to generate PAX2+ cells. All cells died before PAX2 induction occurred. We speculate that the presence of exogenous growth factors combined with bioactive proteins on the ECM were not conducive to differentiation and survival of ES cells.

### ES Cell Derived Metanephric Mesenchyme Cells Grown on Decellularized Kidney Matrices Can Differentiate to Kidney Cells and Develop Tubule Morphology

**Fig. 5:**
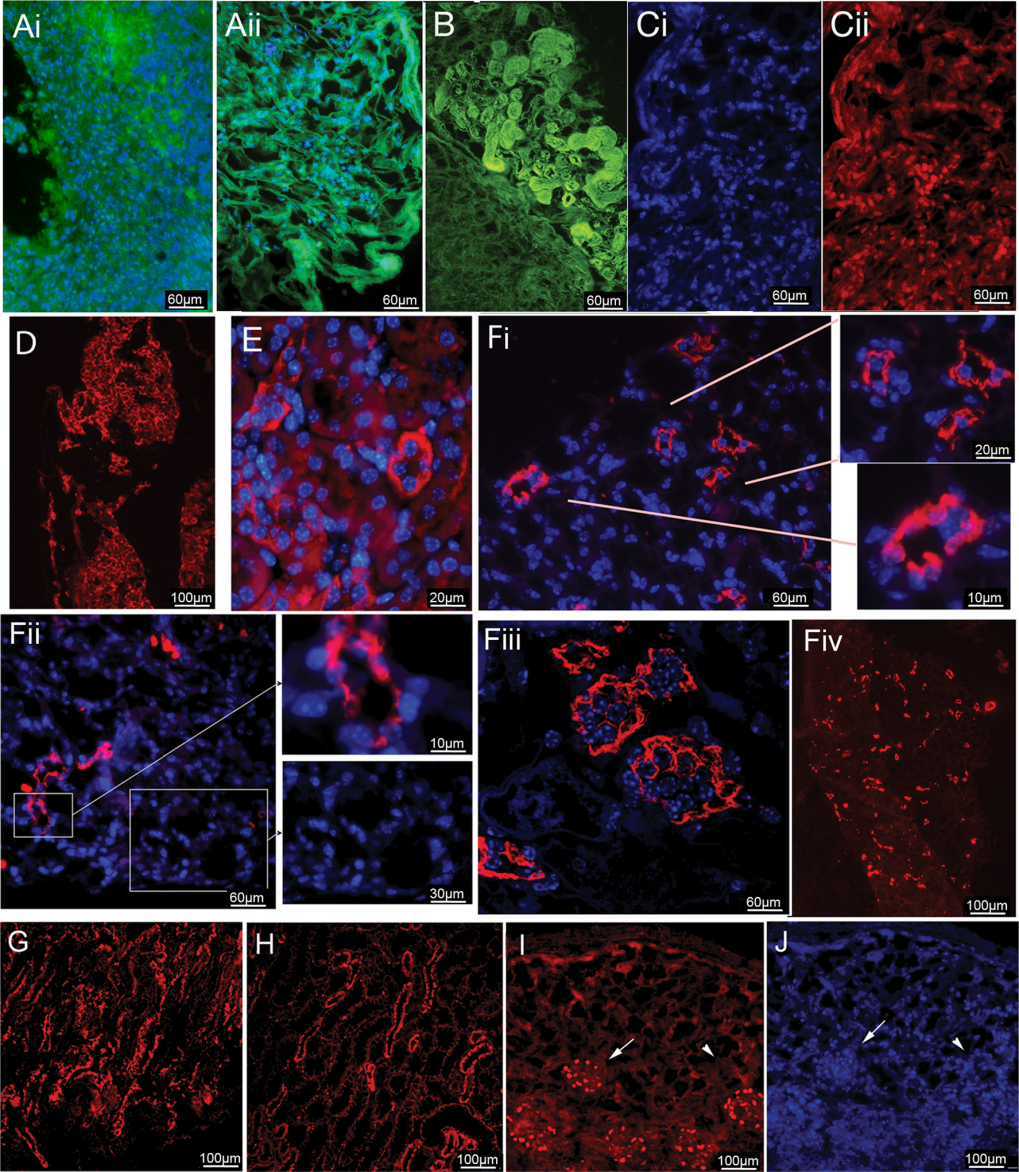
ES cell derived MM cells grown on decellularized kidney matrices can differentiate to kidney progenitor and mature cells and develop tubule morphology. In order to determine if kidney progenitor cells can survive being cultured on acellular kidney matrix, embryonic day 12.5 mouse renal cells were used as a positive control. (**Ai**) After 10 days of culture under the same conditions as the ES cell-derived MM cells, the cell-ECM complexes were fixed and stained for DAPI to highlight the nuclei. The ECM is visible due to autofluorescence (*green*). Most of the ECM was repopulated and cell survival was high. (Aii) The repopulation frequency of the acellular kidney sections using ES cell derived MM cells ranged from 10 to 50%. The cell-matrix construct was stained for the ECM protein Vimentin and the nuclei stain DAPI to highlight cell distribution within the ECM. (**B**) We observed areas of densely populated cells with highly organized tubule structures adjacent to non-cellular areas indicating cell distribution was uneven across the matrix during repopulation. Staining with Collagen type IV indicated the Collagen content was higher in the cellularized area indicating the cells deposit ECM proteins. (**C**) During kidney development PAX2 appears in intermediate mesoderm and condensing mesenchyme. We observed PAX2+ nuclei in cells found in the cortex region of the decelluarized kidney (Ci-DAPI, Cii-PAX2). (**D**) ß-Catenin is expressed during the transition from metanephric mesenchyme to the pretubular stage. ß-Catenin positive cells identify cells differentiating into defined tubule structures. (**E**) Cytokeratin staining of normal mouse kidney outlines tubule structures. (**Fi-iii**) Cytokeratin positive cells defining tubule structures were apparent when the ES cell derived MM cells were grown on the ECM and demonstrated a strong similarity in morphology to normal mouse tubules. (Fiv) Low magnification view of Cytokeratin staining demonstrated that the nascent tubules are found throughout the repopulated ECM. (**G**) KSP, a marker for tubule cells showed similar results to the Cytokeratin staining and was comparable to (**H**) the staining of a normal mouse kidney. (**I**) WT1 is expressed during kidney development in the nuclei of cells during renal vesicle formation, specifically in the prepodocytes during the S-shaped body stage. Expression continues up to mature podocytes. Importantly, WT-1 expression was localized only to the cells that had attached to the glomeruli ECM, indicating that the acellular ECM has regional specificity. Compare WT-1 specific staining in *(I-arrow*) to total cells in (*J-arrowhead*) that are DAPI stained

During kidney development the IM give rise to the nephric duct (ND) which eventually gives rise to the Ureteric Bud (UB), and the MM. The interaction between MM (precursor for the nephron) and the UB (precursor for the Collecting duct and Renal Pelvis) results in the formation of a functional kidney. At this stage of development a single cell suspension isolated from E12.5 mouse kidneys can be cultured in vitro and can undergo differentiation and branching morphogenesis [26, 27]. We hypothesized that if we could induce ES cell derived IM to further differentiate to the equivalent stage of a mouse E12.5 kidney then the cells might survive disassociation and be good candidates to repopulate the acellular kidney. In order to produce E12.5 equivalent kidney cells, monolayers of ES cell derived IM (PAX2+) cells were cultured in modified serum containing medium (SCM) (4% serum in DMEM) supplemented with FGF2 at 0.1–100 ng/ml, +/− BMP-7 at 5–50 ng/ml for 3.5 days. We screened cultures for SIX2+ cells, a marker of MM. While we did achieve some SIX2+ induction with all conditions, the condition with BMP7 (5 ng/ml) and FGF2 (0.1 ng/ml) (M1 medium) or FGF2 alone (0.1 ng/ml) (M2 medium) gave the highest cell survival and the highest yield with 30% of the cells being SIX2+ (Fig. 4Li-ii). Increasing the BMP7 to 25 ng/ml resulted in less SIX2+ cells (Fig. 4Liii). PCR confirmed the induction of MM. *ß-Actin* was used to normalize between experiments. MM positive gene expression included *Pax2, Wt1, Cited 1, Emx2, Sall1* and *Hoxd11*. Because our data indicated that BMP7 is not essential, all further studies were carried out with M2 medium (Fig. 4m). The cultures also contained Cytokeratin + cells indicating that some UB-like cells were present and some cells did not differentiate as they were negative for both SIX2 and Cytokeratin. In order to increase the absolute number of MM cells, we tested using higher plating densities at the mesoderm induction stage. Using 200,000 cells/cm^2^ improved the yield of MM cells overall despite this being sub-optimal for PAX2 induction. Overall, the final protocol to obtain MM cells was a compromise with optimal BRY+ cell yields and PAX2+ cell yields. It is possible that since the cells are not synchronized some cells that are lagging during differentiation get caught up in the last culture stage. The MM cells could be harvested from the plates using accutase and successfully grown on acellular kidney sections using 10% FBS in DMEM (Fig. 5).

E12.5 kidney cells were used to determine the survivability of renal progenitor cells grown on acellular kidney ECM. Cell-ECM complexes were grown for 10 days in 10%FBS/DMEM and resulted in good cell survival indicating the culture conditions would support renal progenitor cells (Fig. 5Ai). For ES cell-derived MM cells the repopulation frequency varied between experiments, but 10–50% repopulation of the acellular kidney was common. Repopulated cells could be found evenly spaced throughout the acellular ECM. The cell-acellular kidney co-cultures were stained with DAPI and Vimentin to highlight cell distribution within the ECM (Figure 5Aii). Staining for Collagen IV revealed that the cells were able to remodel the acellular ECM as the Collagen IV content was higher in the recellularized area. The ability of the cells to remodel the ECM helped to improve the cellular environment and contributed to increased cell survival. Cells also produced highly organized tertiary structures that conformed to the existing tubule ECM (Fig. 5b, compare repopulated area –upper region to unpopulated area-lower region). This observation suggested the cells attached to the existing ECM of the acellular kidney resulting in the relining of the tubule ECM with the new cells. Furthermore, highly organized structures were never observed with non-differentiated ES cells co-cultured with acellular kidneys. MM cell-acellular kidney constructs were stained with non-kidney markers including PDX1, Tubulin III, HNF3β and SOX17 to determine the rate of spontaneous differentiation. All results were negative confirming that no spontaneous differentiation occurred (Data not shown).

During kidney development PAX2 appears during intermediate mesoderm formation, followed by a reduction in the uncondensed mesenchyme but is re-expressed in the nuclei of cells in condensed mesenchyme [28]. We observed PAX2+ nuclei in cells found in the cortex region of the repopulated kidney indicative of the formation of condensing mesenchyme (Fig. 5Ci,ii). In normal kidney development ß-Catenin demarcates the differentiation of metanephric mesenchyme to pretubular stage kidneys. Our observation of ß-Catenin positive cells was in line with the acellular kidney supporting the growth and continued differentiation of ES cell derived MM cells (Fig. 5d) [29]. Cytokeratin staining of a normal mouse kidney (Fig. 5e) was comparable to the Cytokeratin staining demonstrated by the ES cell derived-MM cells that were used to repopulate the acellular kidney. Well-defined tubules are found throughout the repopulated ECM (Fig. 5Fi-iv and insets). KSP is a marker for tubule cells and repopulated areas were observed to have strong KSP staining (Fig. 5g) and had a similar staining pattern as the control kidney (Fig. 5h). WT-1 is important for metanephric mesenchyme maintenance but is also important for proper glomerular development and is expressed in podocytes [30]. WT-1 nuclear protein was observed in cells found in the glomerular region (Fig. 5i) but not in other regions of the repopulated kidney (Compare to DAPI stain Fig. 5j).

## Discussion

Stem cells have the potential to be used in cellular therapy to replace damaged cells within a tissue or to completely replace a damaged organ [31]. One of the main hurdles we face in achieving the goal of stem cell derived organs is replicating the three-dimensional structure. Replicating complex organs such as lung or kidney will require advanced engineering combined with stem cell biology. Furthermore, it is important to produce individual organs that are a match for the patient. Both of these criteria can be met with new technologies. Decellularized organs provide the detailed three-dimensional structure required for building a kidney. Previous studies have demonstrated that the ECM can be preserved after the removal of all cells [14]. Bovine or porcine organs are candidates for acellular substrates as are human organs that are donated but not suitable for transplant. The tissue matching requirement can be achieved by using induced pluripotent stem cells (iPSC). We have made advancements in the understanding of the cell reprogramming process and in producing clinically relevant iPSCs but there are still large variations between different iPSC lines and the differentiation protocols will need to be individualized for each patient sample [32–35]. Therefore differentiation protocols and co-culture systems that include acellular organs are first being developed using embryonic stem cells. We demonstrated this recently using lung [10]. Robust protocols designed using ES cells will then be adapted for patient derived iPSC lines.

The repopulation of an acellular organ or tissue can be done in vitro, prior to transplantation. As well, acellular organs are useful for establishing advanced 3-D tissue culture systems for research on cell migration, adherence and cell-ECM interactions. Organ ECM provides for a natural substrate that is supportive of cell growth. For example, a porcine model for bladder was used to demonstrate the ability of the host cells adjacent to the acellular graft to migrate into the matrix. This indicates the ability of the acellular matrix to attract and support cells during tissue regeneration by providing biological support as well as mechanical support [36]. Acellular tracheas have been transplanted into patients. The trachea from an allogeneic donor was decellularized and then placed in a suspension of the patient’s cells comprised of mobilized endothelial cells, blood cells and mesenchymal stromal cells. Interestingly, the recipient cells used to initially repopulate the trachea were replaced by granulation tissue at 6 months post transplantation and further remodeling resulted in proper reconstitution with new healthy, ciliated respiratory epithelial cells. Although the initial cells used to repopulate the trachea prior to transplant were completely replaced, they conditioned the acellular matrix by remodeling the ECM which resulted in long term tissue repair and not scarring [37, 38]. Clinical trials for bladder reconstruction used acellular matrix from small intestine and not donor bladders, but they have provided a proof of principle for reconstructing organs using acellular matrices [39]. The kidney is a more complex organ therefore inserting a piece of acellular matrix into the kidney and relying on cells adjacent to the construct to repopulate it is not feasible. Even if recipient cells did migrate into the acellular matrix, the likelihood of being able to insert the acellular ECM so it is aligned properly is highly unlikely considering the large number of small diameter tubules. Therefore, a whole, intact acellular kidney would have to be repopulated and cultured in vitro followed by transplantation. It is possible that partial repopulation followed by transplantation may be advantageous as the in vivo environment, which includes the blood supply, should enhance migration, proliferation and differentiation of the cells resulting in a fully functional organ.

Organs respond to damage by initiating a repair program that involves cell proliferation, migration, remodeling of the ECM and re-establishment of function. The ECM contains embedded growth factors that are involved in initiating tissue regeneration and many of these growth factors are also involved in embryo development and organogenesis, thus there are strong similarities between organ repair and organogenesis [40, 41]. The co-opting of some of the embryo development pathways for tissue repair and the observation that repairing tubules undergo a de-differentiation step supports the contention that the ECM could induced the differentiation of nephron progenitor cells to mature nephron cells but could not allow for the induction of pluripotent ES cells to mature nephron cells. The ability of the ECM from adult organs to support the growth and differentiation of tissue progenitor cells has been demonstrated in the lung using ES cell-derived endoderm [10], with heart using fetal derived cardiomyocytes [14] and with the kidney using early post natal renal epithelial cells [1]. The ability of adult ECM to support embryonic, fetal or early post natal cells is due to the ECM’s involvement in adult tissue repair and regeneration and the fact that many of the growth factors used during organogenesis are also used for tissue repair and are present in the adult organ, including the ECM. For example SOX9 is upregulated after acute injury in adult kidneys and inactivation studies in the adult kidney prior to injury demonstrated it is essential for tissue repair [42]. SOX9 is also present during kidney organogenesis and is expressed in the ureteric tip, the ureter mesenchyme and in the S-shaped body [43]. PAX2 shows a similar pattern. It is expressed at the intermediate mesoderm stage of renal development but reappears during renal repair after kidney ischemia-reperfusion [44, 45].

Organogenesis occurs through growth and remodeling of the organ, which in part is controlled by MMPs and TIMPs. The ECM is a reservoir of MMPs and TIMPs and during kidney development growth factors produced by the metanephric mesenchyme work in tandem with the ECM to regulate the expression of MMPs and TIMPs [46]. During kidney repair MMPs and TIMPs are important for remodeling the ECM and establishing normal tissue architecture and thus reducing fibrosis and scaring. Other ECM components, such as Glycosaminoglycan hyaluronan (HA), has a role in regulating branching morphogenesis during kidney organogenesis by enhancing the interaction of HA–and it receptor CD44 at the tips of the new branches as well as promoting cell survival, proliferation and migration [47]. HA and CD44 have these same roles in tissue regeneration after renal ischemia-reperfusion injury. The action of HA is regulated by its molecular weight with small molecules stimulating inflammation while larger molecules stimulate cell proliferation and migration [48]. Although the role of a specific set of proteins, such as the MMPs, maybe different in organogenesis versus regeneration, the fact that they are present and used in both functions provides a rationale for our observation that adult ECM can support fetal stage cells. Furthermore, evidence from the trachea and bladder studies suggests the new cells can remodel the ECM and create a more appropriate environment for their survival. Evidence from our lung study also suggests that the ECM initiates and supports differentiation but the cells remodel the ECM in a similar manner that occurs during organogenesis [10].

Interestingly when the mesoderm and IM differentiation protocols were applied to undifferentiated ES cells grown directly on the ECM, the ES cells did not differentiate and the cells died. This is most likely the result of competing or conflicting signals from the ECM and the additional exogenous factors. We then sought to determine if ES cells pre-differentiated on gelatin coated plates to mesoderm or intermediate mesoderm could continue to differentiate once transferred to acellular kidney substrates. Although it is possible that mesoderm or intermediate mesoderm may have differentiated in response to signals from the acellular kidney we were unable to test this because cells at these stages could not be successfully dissociated. E11.5 kidney MM cells can survive disassociation so we hypothesized that the differentiation of the ES cells to a similar kidney developmental stage would allow us to obtain cells that could be transferred to the acellular kidney. Co-culturing the ES cell-derived MM cells with acellular kidneys allowed us to assess the potential of these cells to become identifiable kidney structures. Our data demonstrated that the adult ECM was compatible with ES cell-derived MM cells and that this population of cells was capable of contributing to tubules and podocytes when co-cultured with acellular kidney ECM. This observation was expected as the ECM plays an important role in inducing the de-differentiation and re-differentiation of kidney cells during the natural repair process [49]. Furthermore, the MM stage kidney cells have an intrinsic ability to differentiate and undergo branching morphogenesis in vitro [26]. So it is possible that the specificity we observed was due to the ECM supporting cell adherence and survival in a location specific manner as the mature kidney cells emerged from the MM.

In this report we present experiments that delineated a strategy for testing potential therapeutic cells on a 3D substrate with the long-term prospect of building a whole bio-artificial organ for transplantation. The data presented here indicates that the acellular kidney provided regionalized factors that are highly instructive, resulting in organized kidney structures within the acellular kidney. The ability to test cells in a three-dimensional culture provided an intermediate step between standard cultures and animal models reducing the cost of cell therapy development. We observed that combining acellular kidneys with a multi-potential population of kidney cells resulted in organized kidney morphology. Therefore the presence of regionalized ECM signals greatly reduced the problem of having to transplant many different mature kidney cell types during the repopulation process. The bioactive role of the ECM makes whole organ manufacturing more realistic.

3D, Three dimensional; ECM, Extra Cellular Matrix; ES cell, Embryonic stem cell; H2K, major histocompatibility loci; IM, Intermediate Mesoderm; MM, Metanephric Mesenchyme; SCM, Serum Containing Medium; SFM, Serum Free Medium.

## Electronic supplementary material

Supplementary Figure 1 Decellularization of mouse kidneys using 0.4% Sodium deoxycholate for 72 h +/− 90 U/ml benzonase for 2 h**.** Whole mouse kidneys were decellularized with 0.4% Sodium deoxycholate for 72 h +/− 90 U/ml benzonase for 2 h and compared to the 0.1% SDS protocol followed by DAPI or H&E staining to detect residual DNA (Blue) and proteins (Red). (A-C) SDS treatment 72 h, (D-F) Sodium deoxycholate treatment 72 h, (G-I) Sodium deoxycholate treatment 72 h + Benzonase treatment 2 h. All samples were stained with DAPI to detect DNA (A, D, G) or H&E stain visualized at low magnification (B,E,H) or high magnification (C,F,I). SDS treatment removed DNA and cell debris (A,B,C), while Sodium deoxycholate treatment alone leaves behind DNA and cell debris (D, E,F) but the residual DNA can be reduced by Benzonase co-treatment as seen by a decrease in blue/purple staining but not red staining (G, H,I). (GIF 1213 kb)

High Resolution Image (TIFF 37742 kb)

Supplementary Figure 2 Immunofluorescence of Control kidney and decellularized kidneys with secondary antibody only controls. Fibronectin, Laminin and Collagen IV were used to stain normal and acellular mouse kidneys. Secondary antibody only was used in all experiments to control for autofluorescence. All primary antibodies are Rabbit IgG. Secondary antibody was an Alexa Fluor-488 anti Rabbit (Molecular Probes #A-11008). DAPI was also included to determine if any residual DNA was left behind after decellularization. Decellularized mouse kidneys did not retain any DNA indicating full decellularization but did maintain ECM proteins, Laminin, Fibronectin and Cytokeratin IV indicating that the decellularization process did not remove these ECM proteins. Secondary only antibody staining is negative indicating the specificity of the IF staining of both control and acellular kidneys. Magnification bars = 40 μm. (GIF 768 kb)

High Resolution Image (TIFF 37315 kb)

Supplementary Figure 3 Growth chamber for whole kidney repopulation. Cell distribution was optimal when vacuum (40 mmHg) was applied to the chamber. (A) A corning glass bottle was modified to work as a kidney repopulation and growth chamber. The bottle is autoclavable and able to withhold a vacuum. Three holes were drilled in the lid to be used as ports for (i) circulating medium and inserting vascular endothelial cells through the artery to repopulate the vasculature, (ii) loading nephron cells through the ureter and (iii) applying vacuum during cell loading and to be used as an air intake during organ culture. (B) The vacuum pulled the renal epithelial cells into the kidney and resulted in an even distribution of cells. (GIF 82 kb)

High Resolution Image (TIFF 2289 kb)
