# Supplementary figures and images for: Acellular Mouse Kidney ECM can be Used as a Three-Dimensional Substrate to Test the Differentiation Potential of Embryonic Stem Cell Derived Renal Progenitors
Source: Stem Cell Rev. 2017 Feb 27;13(4):513–31. doi: 10.1007/s12015-016-9712-2 (PMC5493730; doi:10.1007/s12015-016-9712-2)

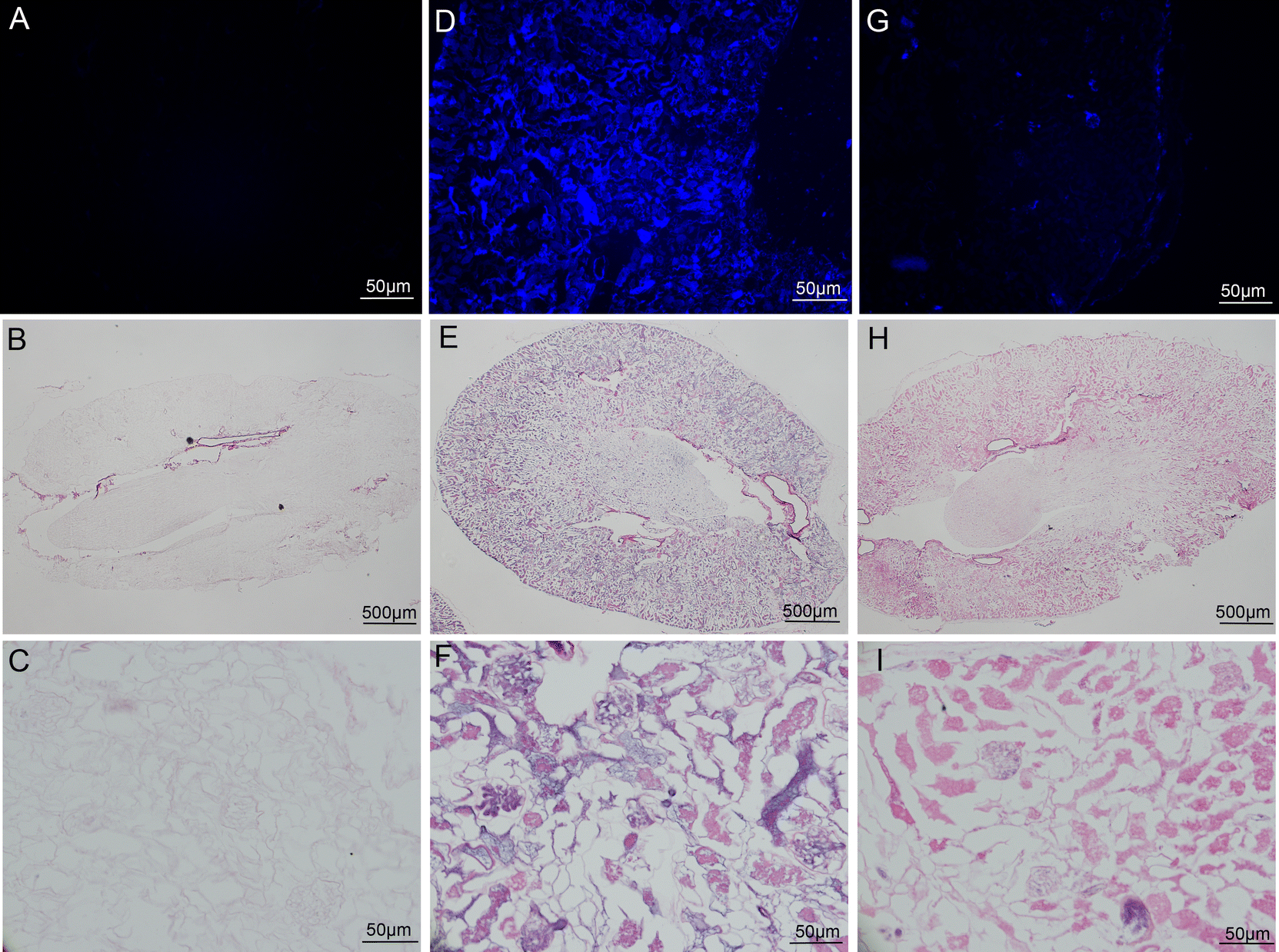

Supplement: Supplementary file 1 — Decellularization of mouse kidneys using 0.4% Sodium deoxycholate for 72 h +/− 90 U/ml benzonase for 2 h. Whole mouse kidneys were decellularized with 0.4% Sodium deoxycholate for 72 h +/− 90 U/ml benzonase for 2 h and compared to the 0.1% SDS protocol followed by DAPI or H&E staining to detect residual DNA (Blue) and proteins (Red). (A-C) SDS treatment 72 h, (D-F) Sodium deoxycholate treatment 72 h, (G-I) Sodium deoxycholate treatment 72 h + Benzonase treatment 2 h. All samples were stained with DAPI to detect DNA (A, D, G) or H&E stain visualized at low magnification (B,E,H) or high magnification (C,F,I). SDS treatment removed DNA and cell debris (A,B,C), while Sodium deoxycholate treatment alone leaves behind DNA and cell debris (D, E,F) but the residual DNA can be reduced by Benzonase co-treatment as seen by a decrease in blue/purple staining but not red staining (G, H,I). (GIF 1213 kb) [file 12015_2016_9712_Fig6_ESM.gif]

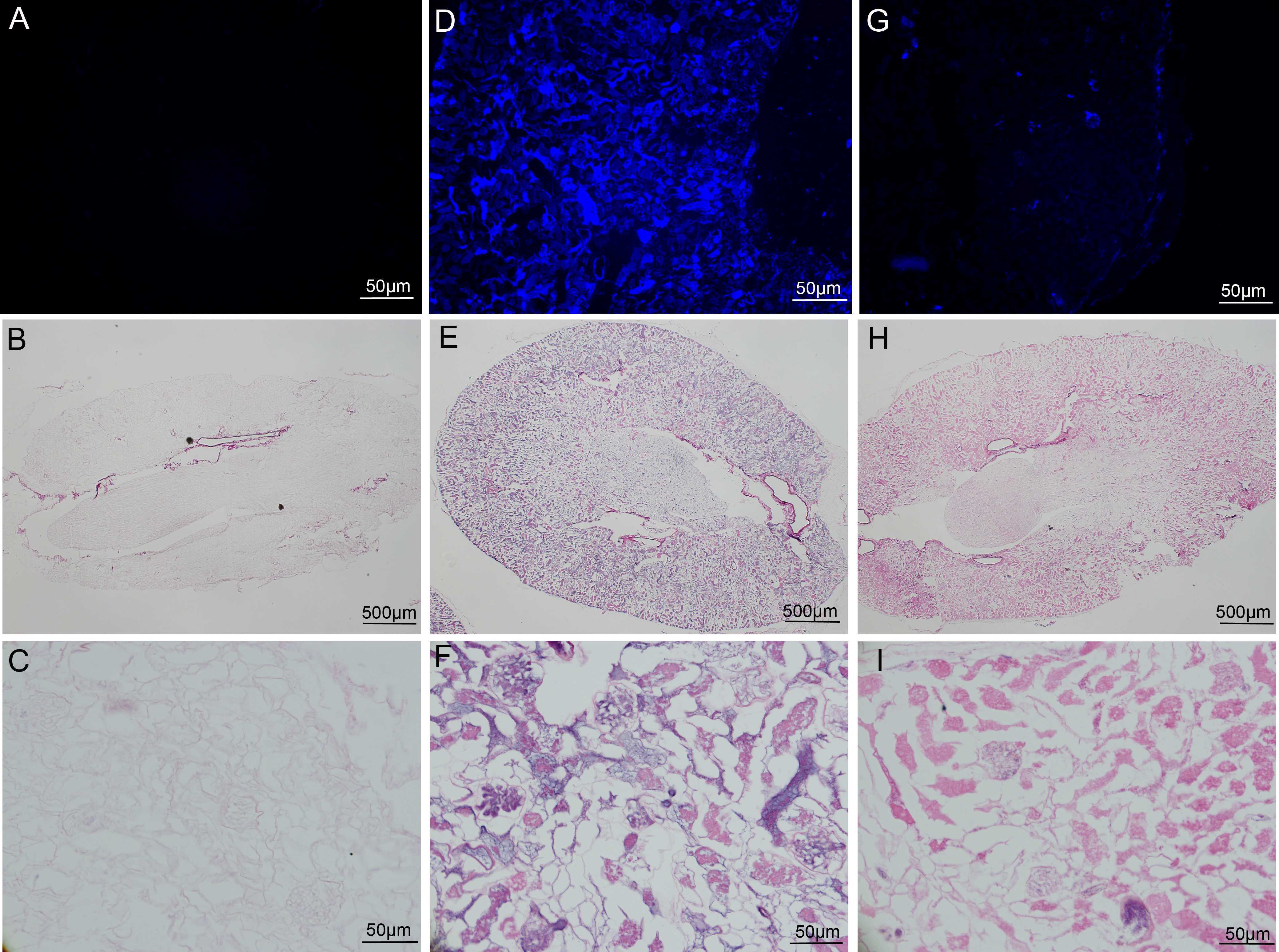

Supplement: Supplementary file 2 — High Resolution Image (TIFF 37742 kb) [file 12015_2016_9712_MOESM1_ESM.tif]

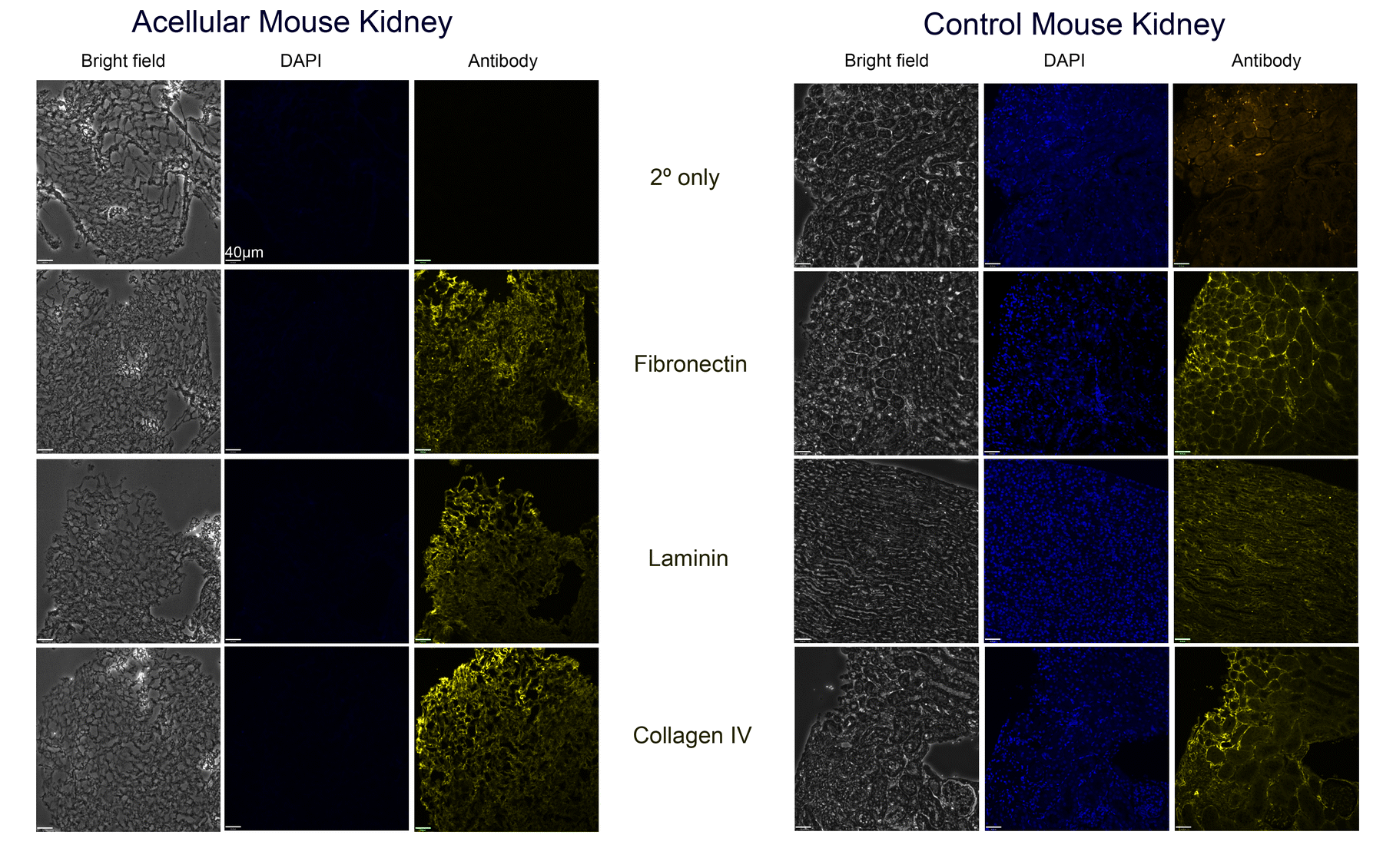

Supplement: Supplementary file 3 — Immunofluorescence of Control kidney and decellularized kidneys with secondary antibody only controls. Fibronectin, Laminin and Collagen IV were used to stain normal and acellular mouse kidneys. Secondary antibody only was used in all experiments to control for autofluorescence. All primary antibodies are Rabbit IgG. Secondary antibody was an Alexa Fluor-488 anti Rabbit (Molecular Probes #A-11008). DAPI was also included to determine if any residual DNA was left behind after decellularization. Decellularized mouse kidneys did not retain any DNA indicating full decellularization but did maintain ECM proteins, Laminin, Fibronectin and Cytokeratin IV indicating that the decellularization process did not remove these ECM proteins. Secondary only antibody staining is negative indicating the specificity of the IF staining of both control and acellular kidneys. Magnification bars = 40 μm. (GIF 768 kb) [file 12015_2016_9712_Fig7_ESM.gif]

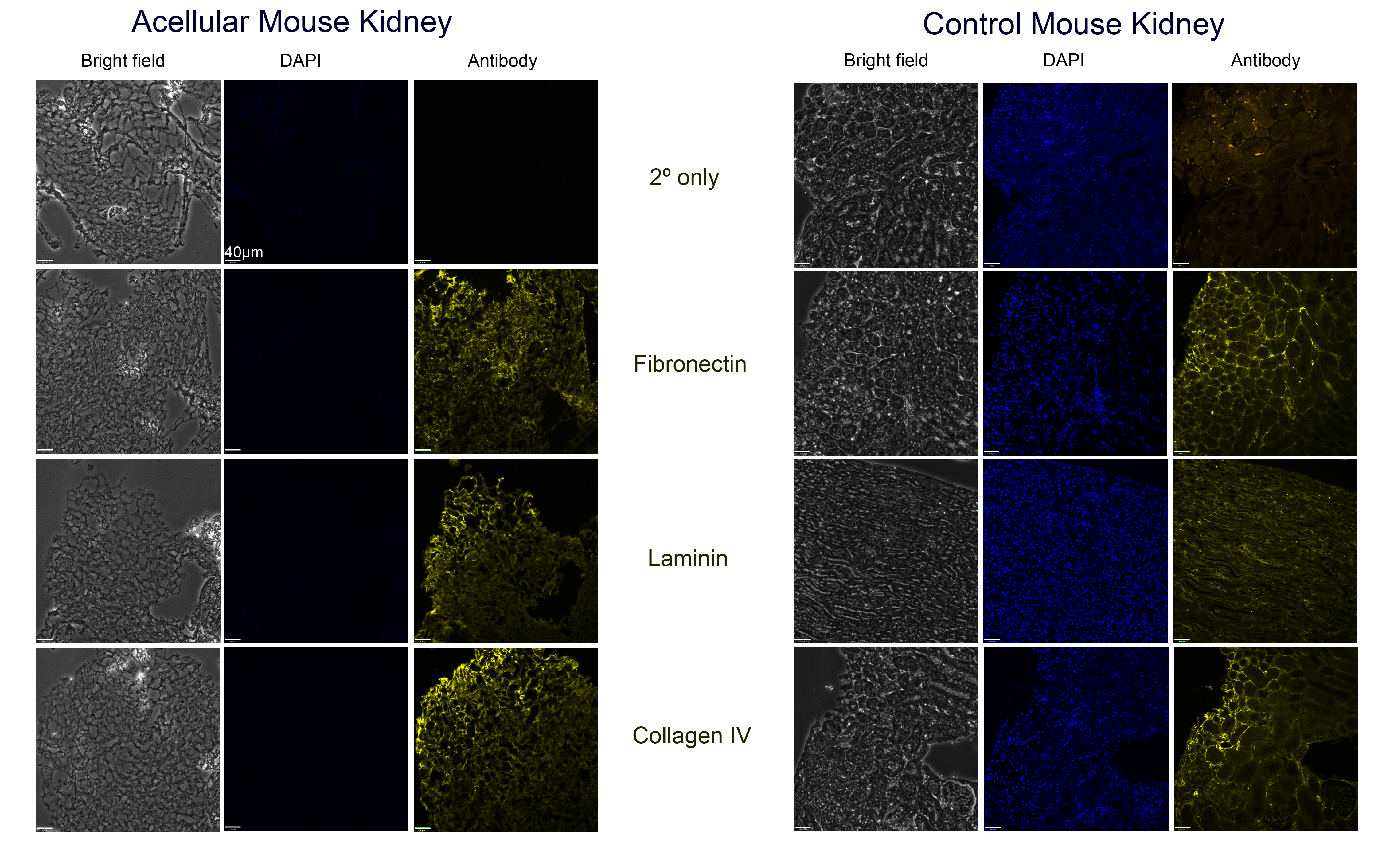

Supplement: Supplementary file 4 — High Resolution Image (TIFF 37315 kb) [file 12015_2016_9712_MOESM2_ESM.tif]

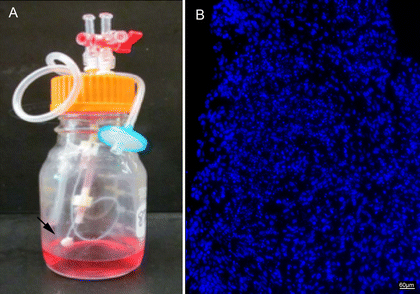

Supplement: Supplementary file 5 — Growth chamber for whole kidney repopulation. Cell distribution was optimal when vacuum (40 mmHg) was applied to the chamber. (A) A corning glass bottle was modified to work as a kidney repopulation and growth chamber. The bottle is autoclavable and able to withhold a vacuum. Three holes were drilled in the lid to be used as ports for (i) circulating medium and inserting vascular endothelial cells through the artery to repopulate the vasculature, (ii) loading nephron cells through the ureter and (iii) applying vacuum during cell loading and to be used as an air intake during organ culture. (B) The vacuum pulled the renal epithelial cells into the kidney and resulted in an even distribution of cells. (GIF 82 kb) [file 12015_2016_9712_Fig8_ESM.gif]

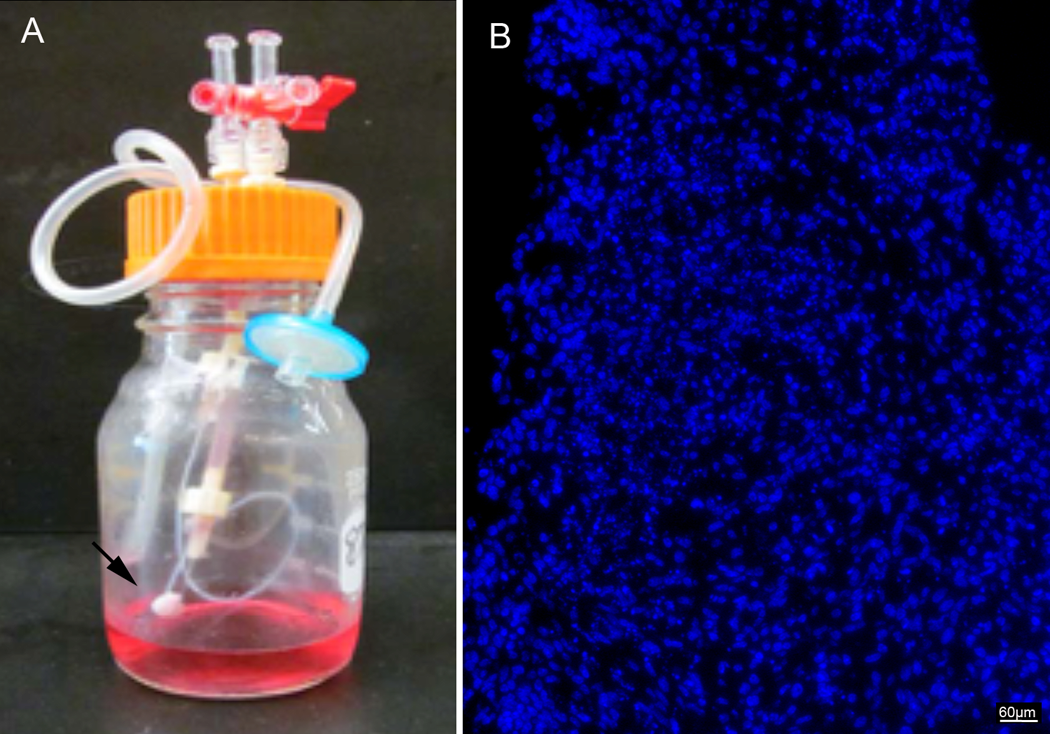

Supplement: Supplementary file 6 — High Resolution Image (TIFF 2289 kb) [file 12015_2016_9712_MOESM3_ESM.tif]
